# Complete Genome Sequence of Methanogenic Archaeon ISO4-G1, a Member of the *Methanomassiliicoccales*, Isolated from a Sheep Rumen

**DOI:** 10.1128/genomeA.00221-16

**Published:** 2016-04-07

**Authors:** William J. Kelly, Dong Li, Suzanne C. Lambie, Jeyamalar Jeyanathan, Faith Cox, Yang Li, Graeme T. Attwood, Eric Altermann, Sinead C. Leahy

**Affiliations:** AgResearch Limited, Grasslands Research Centre, Palmerston North, New Zealand

## Abstract

Methanogenic archaeon ISO4-G1 is a methylotrophic methanogen belonging to the order *Methanomassiliicoccales* that was isolated from a sheep rumen. Its genome has been sequenced to provide information on the genetic diversity of rumen methanogens in order to develop technologies for ruminant methane mitigation.

## GENOME ANNOUNCEMENT

Members of the order *Methanomassiliicoccales* are methylotrophic methanogens first detected in the rumen (1, 2) and subsequently in a variety of other anaerobic environments (3–5). Currently, the only complete genome sequence available for a rumen isolate is from *Thermoplasmatales* archaeon BRNA1 (NCBI reference sequence: NC_020892.1). Here, we report the genome sequence of an isolate from a sheep rumen, which is designated as methanogenic archaeon ISO4-G1 and belongs to a different group within the order *Methanomassiliicoccales* (6, 7).

The complete genome sequence of ISO4-G1 was determined by pyrosequencing 3-kb mate-paired libraries on a 454 GS FLX platform with titanium chemistry combined with reads from a MiSeq 2 × 300-bp sequencing run (Macrogen, South Korea). Pyrosequencing reads were assembled using the Newbler assembler version 2.7 (Roche 454 Life Sciences, USA) and combined with the MiSeq data using the SPAdes assembler version 3.0 (8), resulting in 4 contigs in a single scaffold. Gap closure was managed using the Staden package (9), and gaps were closed using standard PCR techniques with Sanger sequencing. Protein-encoding genes were identified by Glimmer (10), and a GAMOLA/ARTEMIS (11, 12) software suite was used to manage genome annotation. Assignment of protein function to open reading frames was performed manually using results from BLASTp and the COG (Clusters of Orthologous Groups), Pfam, and TIGRFAM databases (13–15).

The genome sequence of methanogenic archaeon ISO4-G1 consists of a single 1,593,503-bp circular chromosome, with a GC content of 55.5%, and 1,501 predicted protein-coding genes representing 92.0% of the genome. The ISO4-G1 genome does not contain plasmid, prophage, or CRISPR sequences. Analysis of the genome suggests that ISO4-G1 relies on hydrogen-dependent methylotrophic methanogenesis to produce energy, with methanol and methylamines as substrates. The complement of methane formation genes is very similar to that reported for “*Candidatus* Methanoplasma termitum” (16). Genes for the production of coenzyme M and tryptophan biosynthesis are missing from the genome. The genome encodes a large number of transporters, including 15 ABC transporters predicted to be involved in Fe3+ or siderophore uptake. Like the other members of the order *Methanomassiliicoccales*, ISO4-G1 has a complete operon (AUP07_0651-654) predicted to encode pyrrolysine biosynthesis together with a specific aminoacyl-tRNA synthetase that enables read-through of the amber stop codon UAG (5, 17). A total of 25 genes encoding pyrrolysine-containing proteins were identified in the ISO4-G1 genome, 9 of which were mono-/di-/tri-methylamine:corrinoid methyltransferases, as reported for other members of the *Methanomassiliicoccales* (5). ISO4-G1 also has a gene (AUP07_0971) predicted to encode a unique pyrrolysine-containing nonribosomal peptide synthase (5,216 amino acid residues) whose function is unknown. Genomic information from this group of organisms will complement genome sequences from other rumen methanogens and will be used to design strategies aimed at reducing methane emissions from ruminant livestock (18).

### Nucleotide sequence accession number.

This whole-genome sequencing project has been deposited at DDBJ/EMBL/GenBank under the accession number CP013703.
